# Comparing times of self-harm presentations to hospital emergency departments in children, adolescents, young adults and adults: a national registry study 2007–2019

**DOI:** 10.1186/s12888-024-05921-x

**Published:** 2024-06-27

**Authors:** David McEvoy, Mary Joyce, David Mongan, Mary Clarke, Mary Codd

**Affiliations:** 1 School of Population Health, Royal College of Surgeons Ireland (RCSI), Beaux Lane House, Mercer Street Lower, Dublin 2, Ireland; 2 National Suicide Research Foundation (NSRF), University College Cork, Western Gateway Building, Cork, Ireland; 3 Centre for Public Health, Queen’s University Belfast, Belfast, Northern Ireland, UK; 4 Department of Psychiatry, Education and Research Centre, Royal College of Surgeons in Ireland, Beaumont Hospital, Dublin 9, Ireland; 5 Physiotherapy and Sports Science, UCD School of Public Health, University College Dublin Belfield, Dublin 4, Ireland

**Keywords:** Self-harm, Self-injury, Emergency department, Presentation time, Age groups

## Abstract

**Purpose:**

The few studies that have explored self-harm presentation times at hospital emergency departments (EDs) – an important factor that can determine if a patient receives a mental health assessment – primarily focus on adult samples. This study examined the times of self-harm presentations to EDs, self-harm methods used, mental health assessments, and admission data across different age-groups.

**Methods:**

Using data from the National Self-Harm Registry Ireland over a 13-year timeframe (2007–2019), this study compared times, days, seasons, methods of self-harm, and admission data for children (8–12 years), adolescents (13–17 years), young adults (18–25 years) and adults (> 25 years).

**Results:**

The majority of the 152,474 self-harm presentations (78.6%) for all ages occurred out-of-hours (outside the standard working hours or in-hours times of 09:00–17:00, Monday-Friday). The four hours before midnight had the highest proportions of self-harm presentations for adolescents (27.9%) and adults (23.1%), whereas the four hours after midnight had the highest proportion of self-harm presentations for young adults (22.9%). The 16:00-midnight timeframe had highest proportion of self-harm presentations in children (52.3%). Higher proportions of patients received a mental health assessment in-hours compared to out-of-hours among young adults (78.2% vs. 73.3%) and adults (76.1% vs. 72.0%). Self-harm presentations were lowest during summer months in children and adolescents.

**Discussion:**

Hospitals should ensure that adequate resources are available for individuals presenting with self-harm, especially in the case of overcrowded EDs, and protocols need to be designed for those presenting with self-harm due to intoxication. In line with national policy, protocols for patients presenting during out-of-hours should be designed that can incorporate services from allied health multidisciplinary teams, social work, addiction services and counselling organisations. Given the lower rates of self-harm during school holidays for children and adolescents, the school environment must be considered in the context of mental health and self-harm public health prevention interventions.

**Supplementary Information:**

The online version contains supplementary material available at 10.1186/s12888-024-05921-x.

## Introduction

Suicide and self-harm are major public health problems globally, especially for young people aged 15–29 years old, for whom suicide is the fourth leading cause of death [1]. Ireland has a suicide rate of 11.0 per 100,000 population in comparison to the European Union’s standardised rate of 10.1 per 100,000 and the United Kingdom’s rate of 7.4 (based on 2017 data) [2]. In 2019, suicide was the leading cause of death in males under 25 years old and the third most common cause in females of the same age in Ireland [2].

Patients who present to hospital emergency departments (EDs) with self-harm are much more likely to die by suicide compared to the general population [3–5]. Estimates amongst studies examining the risk of dying by suicide for those presenting to EDs with self-harm have been found to be up to 50 times more likely than those not presenting to EDs with self-harm [3–5]. While male suicides accounted for the majority (77%) of suicides in Ireland in 2019 [2], the majority (55%) of self-harm presentations to EDs were by females [6].

All patients that present to EDs should receive a mental health assessment from a trained mental health professional [7–9]. A mental health (or psychosocial) assessment is an evaluation of the person’s needs, safety considerations and vulnerabilities that is designed to identify those personal, psychological and environmental or social factors that might explain an act of self‑harm [10]. Furthermore, such assessments should foster building relationships with both the patient and families or other supportive adults and should involve gathering good information on past history and current circumstances to inform a collaborative approach to safety planning [9]. Mental health assessments and appropriate follow-up care for patients presenting with self-harm are essential but previous studies have shown that such assessments are not always universally completed, ranging from 36 to 82% [11–14]. In 2019, 72% of patients attending EDs with self-harm in Ireland received such an assessment [6].

The time of a self-harm presentation at an ED can be an important factor that determines whether a mental health assessment is conducted [11]. Studying the profiles of patients who present at hospital EDs with self-harm, and in particular the times of these presentations, can be informative for hospital management teams to allocate adequate services at critical times.

In a previous scoping review, we showed that the majority of studies with data on times of self-harm presentations at EDs indicated that these presentations mainly occur out-of-hours (i.e. outside 09:00–17:00, Monday to Friday) – in particular, in the hours before and after midnight [15]. For the most part, this scoping review found that time of self-harm presentations tended to be a secondary outcome [15]. In addition, only two of the included studies [16, 17] from this review stratified their data for the time of self-harm presentations at EDs by different age-cohorts: Colman et al. [17] stratified for adults and children and Bergen and Hawton [16] used three age groups [15]. It is possible that, in the other studies that did not stratify for different age-groups, the adult numbers dominated the data, potentially hiding the trends in other age-groups [15]. Furthermore, there was also a dearth of data on the most common weekdays of self-harm presentations at EDs and seasonal data [15].

This study used data from the National Self-Harm Registry Ireland (NSHRI), the world’s first national registry of self-harm presenting to hospital EDs across an entire country [18]. The aim of this descriptive study was to stratify data from the NSHRI database for different age groups and compare the most common times of day, weekdays and seasons for self-harm presentations to EDs. Furthermore, this study compared the following across the different age groups: methods of self-harm; the occurrence of repeat self-harm presentations; whether mental health assessments were carried out; and, admission details.

## Methods

### Study population

This study used data from the NSHRI database for 13 years from 2007 to 2019. NSHRI data have been collected at each ED in the Republic of Ireland since 2006 [18]. The NSHRI uses an internationally-recognised definition for self-harm: namely, “an act with non-fatal outcome in which an individual deliberately initiates a non-habitual behaviour, that without intervention from others will cause self-harm, or deliberately ingests a substance in excess of the prescribed or generally recognised therapeutic dosage, and which is aimed at realising changes that the person desires via the actual or expected physical consequences” [18, 19]. A minimal dataset is used for the purposes of analysis and research [18]. No individual can be identified from the data [18].

Patients were stratified into four age groups: children (aged 8–12 years), adolescents or teenagers (aged 13–17 years), young adults (aged 18–25 years), and adults (aged 26 years or older). Children were defined as persons less than age 13 since this is typically the age when children begin puberty and transition between primary school and secondary school in Ireland [20]. Moreover, onset of self-harm behaviour usually begins in early adolescence between the age of 12 to 14 years [21]. Any data on children aged below age eight were excluded from the NSHRI file in this study due to small numbers. In addition, numbers of participants less than five in categories were hidden in the tables in this study for de-identification purposes.

The definition of adolescence varies across the literature. Youth has been defined as the age-group up to approximately the age of 25 years old [22, 23]. Previously, studies have defined adolescence as the period of life between the start of puberty and the point at which an individual attains a stable, independent role in society; however, the timing of puberty and the transition to adulthood varies across time and cultures [24]. In Ireland, it has been recommended that the age range for eligibility for child and adolescents mental health services (CAMHS) be increased to 25 years to improve the continuity of care [25]. Moreover, young people typically leave secondary school, to move onto third level education or work, around the age of 18 years. With all of these considerations in mind, adolescents were defined as being aged 13–17 years and young adults as being aged 18–25 [22].

### Data items

This study used NSHRI data on the sex and age of the patient. The primary outcomes for this study were the times of day, weekdays and months of self-harm presentations. Hourly time frames were analysed and the 24-hour clock was also split into four-hour time frames beginning at midnight. Following on from the study conducted prior to this one [15], in-hours (or standard working hours) were defined as the hours of 09:00–17:00 on Mondays through to Fridays and excluded weekends. Out-of-hours were defined as outside of these hours.

The secondary outcomes included methods of self-harm; the occurrence of repeat self-harm presentations; whether mental health assessments were carried out; and, admission details. Methods of self-harm are coded in the NSHRI database using the WHO International Classification of Diseases (ICD-10) codes for intentional self-injury [26]. For the purposes of this study, these codes were collapsed into six categories for methods of self-harm: drug overdoses only; self-cutting only; overdoses and self-cutting; attempted hanging only; attempted drowning only; and, other methods. Methods of self-harm under ‘other’ referred to a myriad of self-harm methods such as ingesting chemicals and noxious substances; crashing a motor vehicle; use of petroleum products, other solvents or their vapours; using alcohol; use of a blunt (non-sharp) object; jumping from a height; jumping in front of or lying in front of a moving object; use of fire or flames; and, use of rifles, shotgun and large firearm discharge etc.

### Data analysis

After stratifying our data into the four age-groups, the percentage proportions were calculated for the aforementioned primary and secondary outcomes for each age-group. With respect to time, this study examined the differences between the age groups using both hourly time frames, four-hour time frames, and attendances during in-hours and out-of-hours. The analyses mentioned were also conducted for males and females within each of the age groups. Moreover, within each of the four age groups, the chi-square test was used to test statistical differences for whether the patient received a mental health assessment in-hours compared to out-of-hours. Given that we conducted four hypothesis tests, we applied the Bonferroni correction to α = 0.05 and tested at α = 0.0125 level of significance. In very large sample sizes, even small differences between the groups can lead to statistically significant results rendering the practical significance of the standard *p*-values meaningless. Therefore, effect sizes (phi coefficient) were also calculated to quantify the magnitude of the differences between the groups. All analyses were completed using R.

## Results

There were 152,474 self-harm presentations involving *n* = 90,333 individuals made to EDs in Ireland between 2007 and 2019. Descriptive data for the primary and secondary outcomes of this study, with stratifications for the four age groups, are presented in Table 1. Further stratification analysis of the age groups for males and females can be viewed in the supplementary material.

**Table 1:** Descriptive data of self-harm presentations for patient sex, time of day for presentations, out-of-hours timeframe, method of self-harm presentation, whether it was a repeat self-harm presentation, and mental health assessment and admission details

	Children (age 8–12)	Adolescents (age 13–17)	Young Adults (age 18–25)	Adults (age > 25)	Total (all ages)
**n (%)**	656 (0.4)	16,587 (10.9)	38,989 (25.6)	96,242 (63.1)	152,474 (100.0)
	**n (%)**	**n (%)**	**n (%)**	**n (%)**	**n (%)**
**Repeat self-harm presentations**					
Yes	40 (6.1)	4,120 (24.8)	14,926 (38.3)	43,056 (44.7)	62,142 (40.8)
No	616 (93.9)	12,467 (75.2)	24,063 (61.7)	53,186 (55.3)	90,332 (59.2)
**Sex**					
Male	277 (42.2)	4,820 (29.1)	18,900 (48.5)	45,570 (47.3)	69,567 (45.6)
Female	379 (57.8)	11,767 (70.9)	20,089 (51.5)	50,672 (52.7)	82,907 (54.4)
**Time of Presentation**					
00:00–03:59	67 (10.2)	3,260 (19.7)	8,919 (22.9)	19,649 (20.4)	31,895 (20.9)
04:00–07:59	10 (1.5)	853 (5.1)	5,148 (13.2)	8,900 (9.2)	14,911 (9.8)
08:00–11:59	77 (11.7)	1,458 (8.8)	3,269 (8.4)	8,846 (9.2)	13,650 (9.0)
12:00–15:59	159 (24.2)	2,721 (16.4)	5,806 (14.9)	16,586 (17.2)	25,272 (16.6)
16:00–19:59	172 (26.2)	3,661 (22.1)	7,321 (18.8)	20,065 (20.8)	31,219 (20.5)
20:00–23:59	171 (26.1)	4,634 (27.9)	8,526 (21.9)	22,196 (23.1)	35,527 (23.3)
**In-hours versus out-of-hours**					
In-hours (09:00–17:00, Monday to Friday)	228 (34.8)	3,799 (22.9)	7,374 (18.9)	21,261 (22.1)	32,662 (21.4)
Out-of-hours	428 (65.2)	12,788 (77.1)	31,615 (81.1)	74,981 (77.9)	119,812 (78.6)
**Method of self-harm**					
Drug overdose only	184 (28.0)	9,238 (55.7)	20,981 (53.8)	61,159 (63.5)	91,562 (60.1)
Self-cutting only	225 (34.3)	3,851 (23.2)	8,777 (22.5)	15,668 (16.3)	28,521 (18.7)
Overdose & self-cutting	15 (2.3)	1,031 (6.2)	2,478 (6.4)	3,720 (3.9)	7,244 (4.8)
Attempted hanging only	109 (16.6)	732 (4.4)	1,891 (4.9)	4,171 (4.3)	6,903 (4.5)
Attempted drowning only	5 (0.8)	132 (0.8)	822 (2.1)	2,368 (2.5)	3,327 (2.2)
Other	118 (18.0)	1,603 (9.7)	4,040 (10.4)	9,156 (9.5)	14,917 (9.8)
**Mental health assessment conducted (** ***n*** = 81,481)*					
Yes	335 (71.1)	7,032 (69.0)	13,722 (66.4)	32,950 (65.7)	54,039 (66.3)
No	80 (17.0)	2,335 (22.9)	4,737 (22.9)	12,224 (24.4)	19,376 (23.8)
Refused	< 5 (< 2.0)	126 (1.2)	729 (3.5)	1,806 (3.6)	2,663 (3.3)
Unknown	54 (11.5)	696 (6.8)	1,482 (7.2)	3,171 (6.3)	5,403 (6.6)
**Admission details**					
Admitted to a ward	312 (47.6)	6,064 (36.6)	7,952 (20.4)	26,419 (27.5)	40,747 (26.7)
Admitted to psychiatry	7 (1.1)	449 (2.7)	3,127 (8.0)	9,939 (10.3)	13,522 (8.9)
Refused admission or left against medical advice	9 (1.4)	984 (5.9)	5,757 (14.8)	14,806 (15.4)	21,556 (14.1)
Not admitted	328 (50.0)	9,090 (54.8)	22,153 (56.8)	45,078 (46.8)	76,649 (50.3)

Note: * only data available from 2013–2019

The majority of the self-harm presentations were from adults aged over 25 years (63.1%); followed by young adults (25.6%); then, adolescents (10.9%); and, children (aged 8–12) accounted for less than 1% of these ED presentations. For children, 6.1% of self-harm presentations were repeat self-harm presentations and this percentage increased through the age-groups to 24.8% in adolescents, 38.3% in young adults, and 44.7% in adults. The proportion of males to females varied across age groups; however, for adolescents the proportion of female self-harm presentations versus male was larger (70.9% vs. 29.1%) than in the other three groups. A similar observation was made for children although to a lesser degree (57.8% vs. 42.2%). There were only slightly higher female percentages in both the young adult and adult age groups.

### Time of self-harm presentations

The highest proportion of self-harm presentations (23.3%) occurred during the 20:00 to midnight time-frame when all age groups were pooled together. The lowest proportion for all age groups pooled together was 08:00 to midday (9%). The most common time for self-harm presentations involving children occurred between midday and midnight, with the highest proportion (52.3%) of self-harm presentations occurring between 16:00 and midnight. The highest proportion (27.9%) for adolescents occurred during the 20:00 to midnight time-frame and the lowest proportion (5.1%) was during the 4:00–8:00 timeframe. Similarly, the highest proportion (23.1%) for adults also occurred during the 20:00 to midnight time-frame, with the lowest numbers of presentations occurring during the period from 04:00 to midday. The highest proportion of young adult self-harm presentations (22.9%) was during the midnight to 04:00 timeframe and the lowest proportion was from 08:00 to midday.

Pooling all age groups together, the majority (78.6%) of all self-harm presentations occurred out-of-hours (outside 09:00–17:00, Monday to Friday). While more self-harm presentations for children did occur out-of-hours (65.2%), this was lower in comparison to the other three age-groups. The proportions of adolescent, young adult, and adult self-harm presentations during out-of-hours were 77.1%, 81.1% and 77.9%, respectively.

**Fig. 1:**
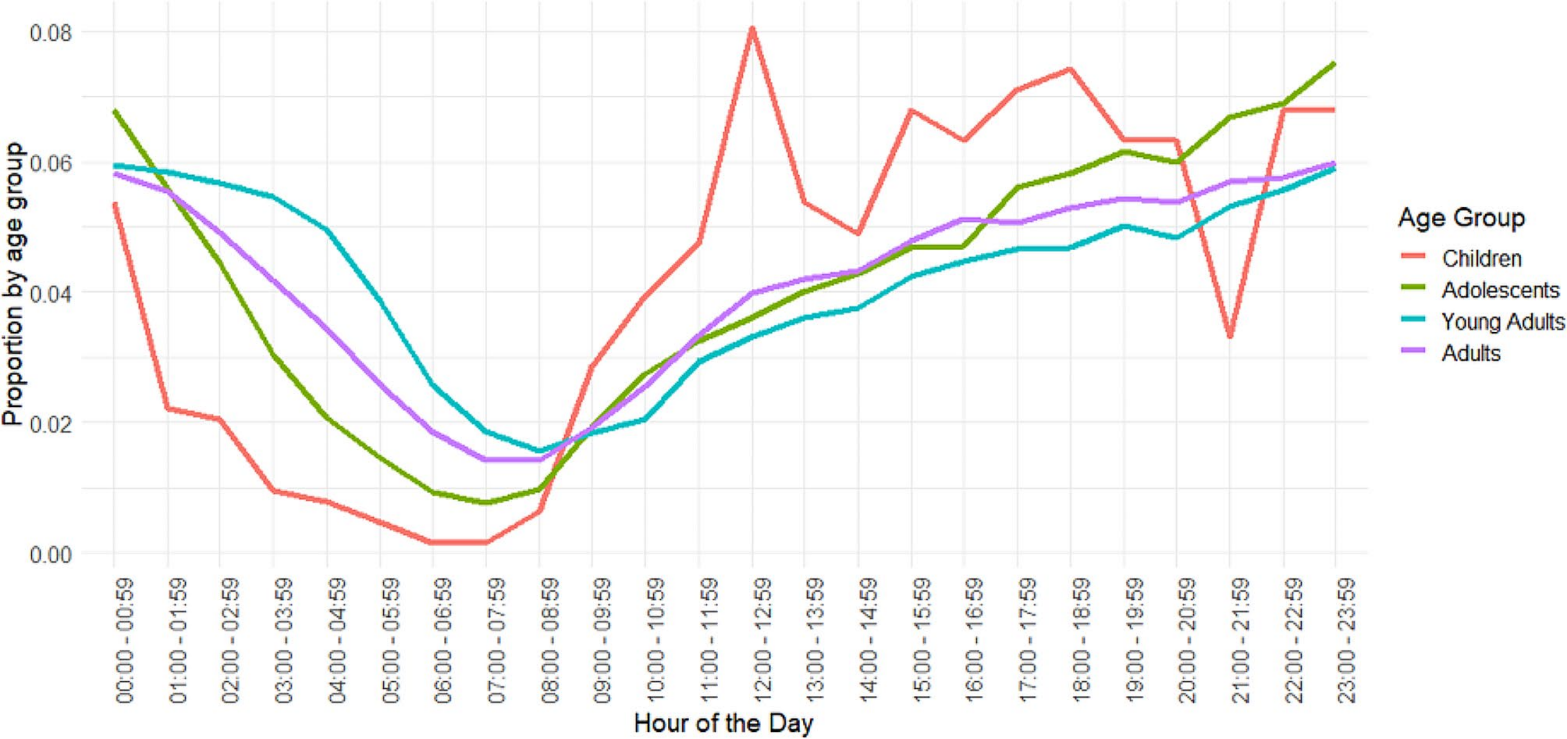
Proportions of self-harm presentations for the four age groups for times of day

The proportions of self-harm presentations for each hour, stratified for each age group, are displayed in Fig. 1. For children, the peak hour for self-harm presentations was between midday and 13:00. We can also see the higher proportions of self-harm presentations in this age cohort after midday in comparison to before midday. The peak hour for adolescents was from 23:00 to midnight but was also high from 22:00–23:00 and from midnight to 01:00. There was not a clearly defined peak hour for adults and young adults; rather, the peak times were in the three to four hours before and after midnight.

#### Weekdays of self-harm presentations

**Fig. 2:**
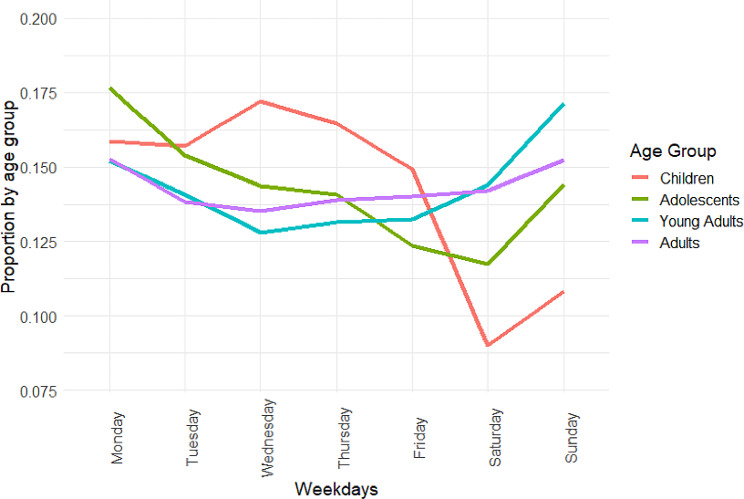
Proportions of self-harm presentations for the four age groups for weekdays

The proportions for self-harm presentations for children were typically higher on weekdays, particularly on Wednesdays (17.2%) and Thursdays (16.5%), and lower during weekends. See Fig. 2. For adolescents, the proportion of self-harm presentations were highest on Mondays (17.6%) and decreased as the week went on, with the lowest proportion on Saturdays (11.7%), but rose again on Sundays. In contrast, young adults had the highest proportions on Sundays (17.2%) and Mondays (15.2%), with lower proportions mid-week. Adult self-harm presentations also followed this trend, but was more evenly spread across the week in comparison to young adults.

#### Months of self-harm presentations

**Fig. 3:**
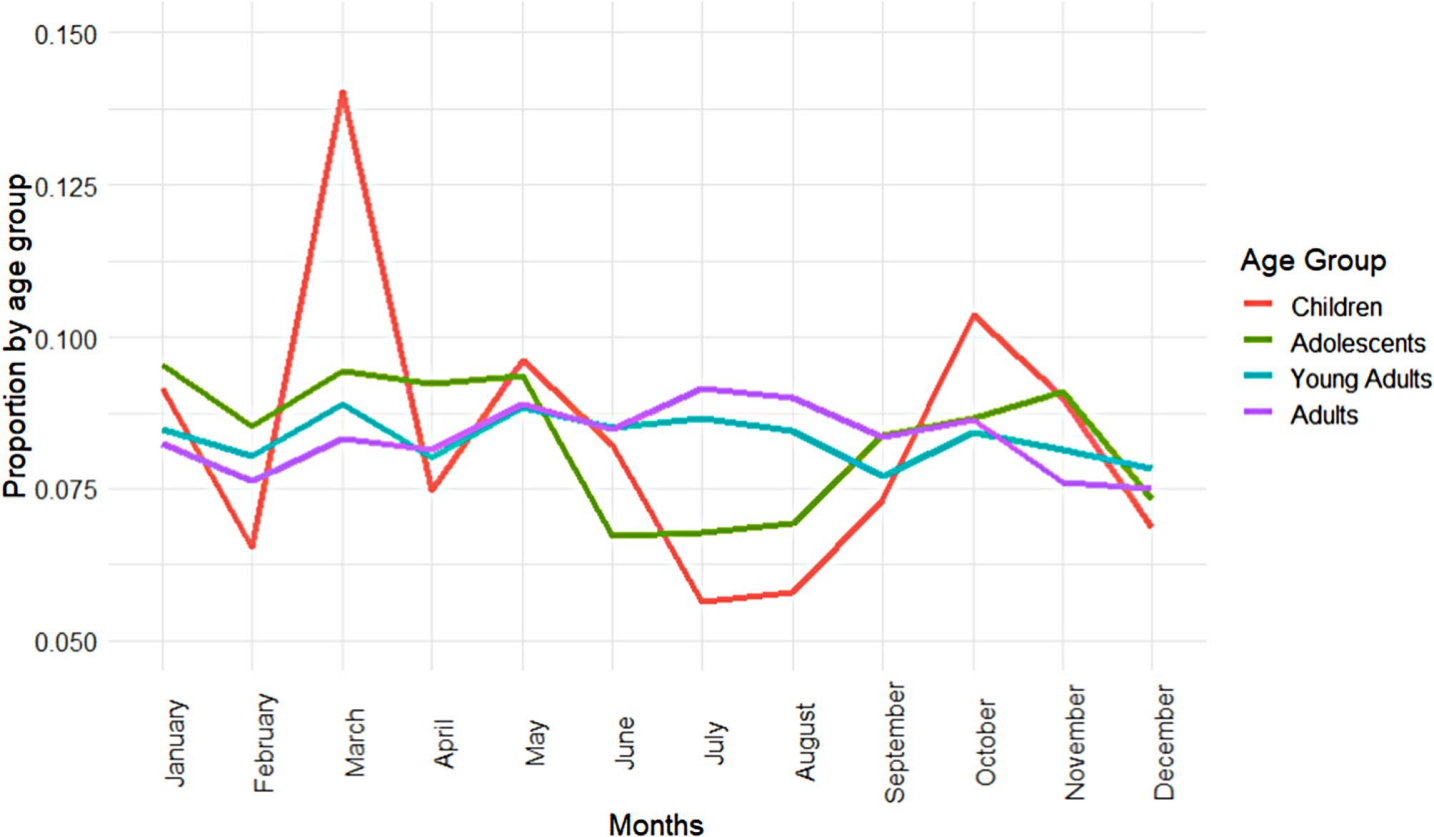
Proportions of self-harm presentations for the four age groups for months

The highest proportions of self-harm presentations for children occurred during March (14.0%) and October (10.4%), whereas the lowest proportions of self-harm presentations for children occurred during July (5.6%) and August (5.8%). See Fig. 3. Adolescents’ self-harm presentations proportions were highest in January (9.5%) and March (9.4%), and similar to children, the proportions of self-harm presentations were lowest in the summer months during June (6.7%), July (6.8%) and August (6.9%), and was also low during December (7.3%). The proportions for self-harm presentations in young adults and adults were similar across the 12 months, with a slight increase in the proportions during the summer months for adults. The highest proportion of young adults was in March (8.9%) and May (8.8%). The highest proportion for adults was in July (9.2%).

#### Methods of self-harm

**Fig. 4:**
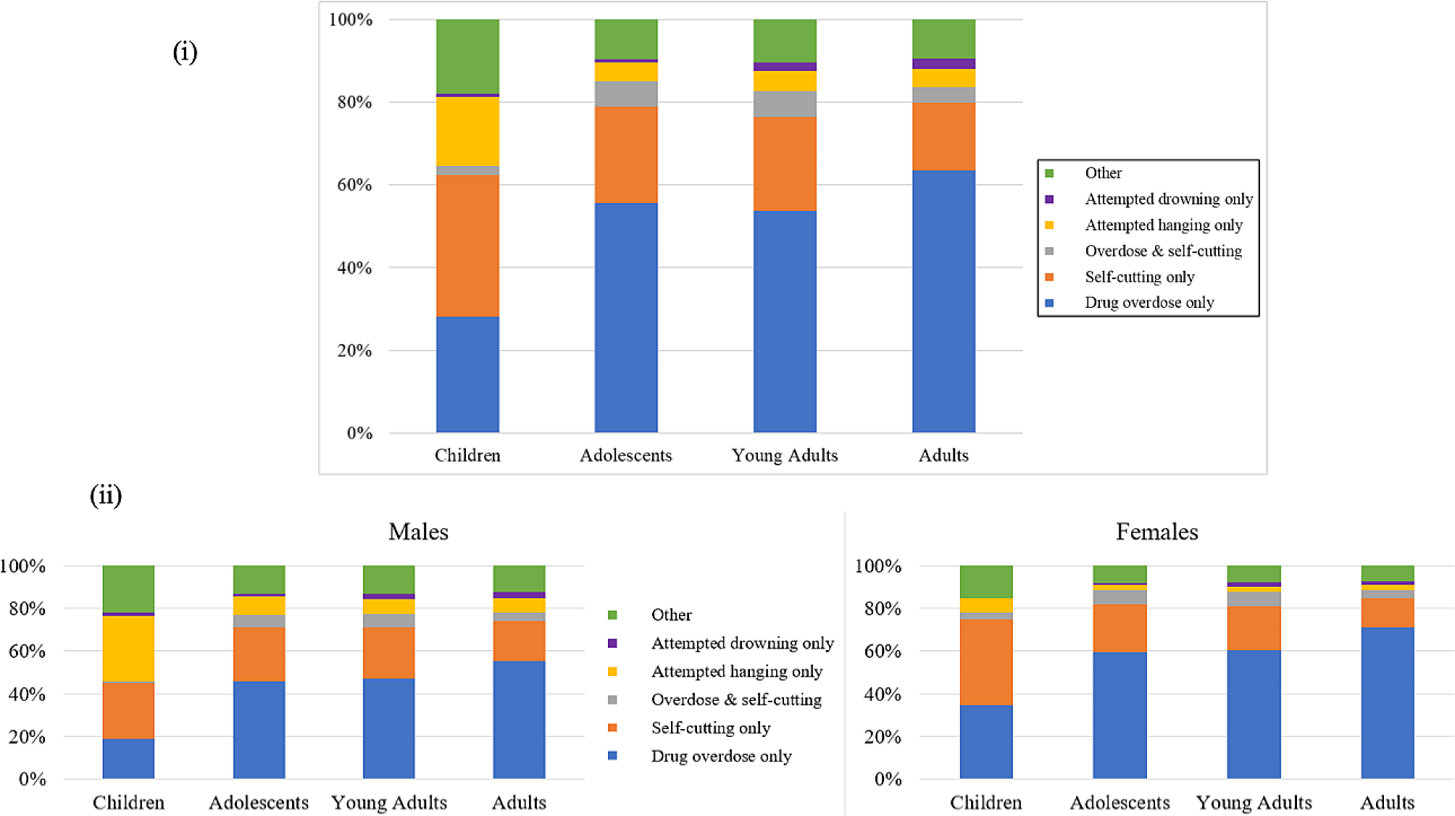
(i) Proportions of methods used in self-harm presentations for the four age groups (ii) Proportions of methods used in self-harm presentations for the four age groups stratified for sex

For adolescents, young adults and adults, drug overdoses accounted for the majority of self-harm presentations with proportions of 55.7%, 53.8% and 63.5%, respectively for the three age-groups. See Fig. 4 (i). For the same three age-groups (in the same order), the next biggest proportion of self-harm presentations were self-cutting presentations, with proportions of 23.2%, 22.5% and 16.3%, respectively. Presentations with a combination of these two methods were lower in adults (3.9%) compared to adolescents and young adults (both over 6%). Other methods of self-harm accounted for approximately 10% and attempted hanging accounted for 4–5% in each of these three age-groups. Attempted drowning was lower in adolescents (less than 1%) compared to over 2% in both young adults and adults.

While these three age-groups had similar proportions across the different methods of self-harm, they were quite different for children. For children, self-cutting was the most common method (34.3%), which was followed by drug overdoses (28%). Attempted hangings in children self-harm presentations accounted for 16.6% and other methods accounted for 18% of self-harm presentations in children.

When we further stratified data on methods of self-harm by sex (see Fig. 4 (ii) and the supplementary material), attempted hanging was the most common method in male children (nearly 30.7% versus less than 7% in females), whereas self-cutting was the most common method in female children (40.1% compared to 26.4% in male children). Female children had higher proportions of drug overdoses (34.8%) compared to male children (18.8%). For each age group, females had higher proportions of drug overdoses in comparison to males. The highest proportion of drug overdoses in sex and age-groups was in female adults (71%). Males had higher proportion of more lethal methods of self-harm such as attempted hangings, attempted drownings and other methods compared to females across all the age groups.

#### Mental health assessments and admission details

The percentages for those receiving a mental health assessment were highest in children and adolescents (71.1% and 69.0%, respectively) compared to young adults and adults (66.4% and 65.7%, respectively). The highest proportion (3.6%) for those refusing a mental health assessment was in adults. Likewise, the highest proportions of those refusing admission or leaving against medical advice were in both young adults and adults (14.8% and 15.4%, respectively). For all four age groups, there were higher proportions that received a mental health assessment in-hours compared to out-of-hours. (See supplementary material Table 2.) These proportions, though not substantially different, were only found to be statistically significant in both the young adult and adult groups. In addition, the proportional variation in those receiving and not receiving mental health assessments in these two age groups was small, as indicated by the small effect sizes. In young adults, 78.2% of those who attended in-hours received a mental health assessment, compared to 73.3% of those who attended out-of-hours. In adults, 76.1% of those who attended in-hours received a mental health assessment, compared to 72% of those who attended out-of-hours.

## Discussion

This study found that most self-harm presentations (78.6%) occurred out-of-hours (outside 9:00–17:00, Monday to Friday), with particularly high proportions in the four hours before and after midnight. This was not the case for children, for whom the rates of self-harm presentations peaked from 16:00 in the evening until midnight. Previous studies have also demonstrated that most self-harm presentations occur out-of-hours, with the peak times usually in the hours before and after midnight [15]. Children presented more commonly midweek and less often during weekends, whereas the proportions of young adult and adult self-harm presentations were highest on Sundays and Mondays and lower midweek. Adolescent presentations were highest on Mondays and lowest on Saturdays. Higher proportions of patients received a psychiatric review during in-hours compared to out-of-hours, though this was found to be statistically significant only in young adults (78.2% vs. 73.3%) and adults (76.1% vs. 72.0%).

Approximately 3.5% of young adults and adults refused a mental health assessment in comparison to less than 2% in the adolescent and children cohorts. Furthermore, approximately 15% of young adults and adults refused admission or left against medical advice in comparison to only 5.9% and 1.4% in adolescents and children, respectively. Alcohol involvement in the self-harm presentation is one factor that may account for whether the patient was discharged without a mental health assessment [8]. Previous research has found that self-harm presentations involving alcohol peak after midnight and on Sundays and Mondays in comparison to presentations not involving alcohol, which tend to be more evenly spread out across the week and with a less accentuated peak between 18:00 and midnight [27]. Hence, it is likely that many of the self-harm presentations accounting for the lower proportions of young adults and adults not receiving mental health assessments are due to the involvement of alcohol [8]. More comprehensive addiction pathways for patients presenting during out-of-hours have previously been recommended for patients presenting to EDs with self-harm [8].

Appropriate pathways to voluntary counselling services have also been recommended for patients who present to EDs with self-harm but differences in referrals to these agencies have been found for those presenting in-hours and out-of-hours [8]. Indeed, the lack of availability of allied services, such as multidisciplinary teams and social work support out-of-hours may be another reason for the lower percentages of mental health assessments being conducted [28]. Overcrowding is another issue facing Irish EDs, which is associated with poorer patient outcomes including higher mortality rates [29]. The provision of care for patients presenting to EDs with self-harm and the availability of specialised mental health resources varies across different hospitals [30]. One of the recommendations from Ireland’s national mental health policy, *Sharing the Vision*, is that there should be continued investment in, and implementation of, a national critical care programme for the assessment and management of patients presenting to EDs following self-harm [25]. Health service managers should strive to ensure that adequate resources for patients with self-harm are available for those who need them out-of-hours. In addition to ensuring availability of an in-depth mental health assessment (for example through appropriate multidisciplinary staffing of psychiatric services out-of-hours), this may also involve working closely with other local agencies including voluntary sector organisations (such as crisis counselling).

The data from the current study justify the recommendations from the most recently published National Clinical Programme for Self-Harm and Suicide-related Ideation (NCPSH) [9]. NCPSH recommends that mental health assessments are provided to patients presenting with self-harm at EDs regardless of the time [9]. It also mentions that each service should ensure that a procedure is in place to ensure the handover of details of all patients who present out-of-hours and that each patient’s GP should receive immediate secure communication on the patient’s presentation and emergency plan [9]. This is particularly relevant given that the majority (64.4%) of patients that leave the ED after a self-harm presentation without admission to either a general or psychiatry ward. In addition, the NCPSH mentions that each patient should receive a follow-up phone call from a mental health professional, such as a clinical nurse specialist, within 24 h of discharge from ED [9]. Developing crisis assessment teams and suicide crisis assessment nurses by mental health services in Ireland who will work with GPs have further been recommended [9]. Given the association between alcohol and self-harm discussed previously [8], the NCPSH further recommends opportunistic mental health assessment screening for those presenting at EDs at risk of alcohol or substance misuse [9].

Males made up a smaller proportion of self-harm presentations at EDs compared to females, especially in adolescents, while paradoxically accounting for the vast majority of deaths by suicide [2]. As in other studies, this study also found that more lethal means of self-harm, such as attempted hanging or drowning, were more common in males [27]. In fact, the percentage for attempted hangings was the greatest proportion for any method in male children - nearly 31% in male children compared to less than 6.3% in female children. The proportions of those not receiving a mental health assessment or refusing admission or leaving against medical advice were slightly higher in males also compared to females. (See supplementary material). Therefore, males presenting to EDs with self-harm are potentially a high-risk group.

For adolescents and even more so for children, the rates of self-harm were lowest during the summer months. For adolescents, the rates were lowest in June, July and August; whereas for children the rates were lowest for July and August. These align with secondary school and primary school holidays in Ireland, respectively. In addition, the rates were lower in December, during which schools have a two-week Christmas break. In a Canadian study, Colman et al. also found that self-harm presentations were lower for children during summer months [17]. There has also been some evidence to show that both suicide rates and mental health presentations to EDs are lower during summer months in the United States [31]. Furthermore, children’s rates of self-harm ED presentations peaked during weekdays and were lowest on weekends. For adolescents, self-harm ED presentations peaked on Mondays and fell as the weekdays went on, with the lowest rates on Saturdays and rising again on Sundays.

School staff are often the ones to identify young people exhibiting self-harm behaviour [32], and so the lower rates of self-harm during the school holidays may be attributed to the decrease in detection. On the other hand, there are many risk factors for self-harm in young people that involve the school setting (such as bullying, social contagion of self-harm behaviour in peer groups, truancy, and low academic performance) [33], and this may also account for the higher levels of self-harm during the school months. While school may not necessarily always be a causative factor for self-harm in young people, it must be considered in the context of mental health and self-harm public health prevention interventions in children and adolescents. *Sharing the Vision* also sets out that every school in Ireland should have a dynamic wellbeing promotion process [25]. Furthermore, *Sharing the Vision* recommends that a liaison process should be in place between schools, mental health services, GPs, primary care services, and specialist mental health services [25].

For adults, the seasonal data are similar to the existing literature with higher self-harm presentations occurring in the summer months [17, 34–37]. There has been some evidence to suggest that higher temperatures are associated with small increases in hospitalisations due to self-harm [38]. To our knowledge there has been no credible reason for this phenomenon. Previously, we conjectured that alcohol, longer days, idleness, or loneliness during the holiday season could be factors contributing to the peak rates in self-harm presentations at EDs in summer months [15].

### Strengths and limitations

Using data from a national self-harm registry of all ED presentations for this study is unique in global terms and is a major strength to this study. Moreover, we were able to use 13 years of data. On the other hand, hospital-presenting self-harm data does not accurately describe self-harm in community settings where the individuals do not necessarily present at a hospital ED. Hence, the results of this study should be interpreted with this in mind. The rate of community self-harm has been shown to vastly outnumber hospital-presenting self-harm [39]. An iceberg model of self-harm has been used to describe this phenomenon whereby hospital presenting self-harm is visible above the surface but community self-harm is vastly bigger below [39]. While we found that overdosing was the most common method for self-harm in adolescents and young adults for example, it has been hypothesised that self-cutting would actually be the most common method in the community setting [40].

Data on when the self-harm act occurred prior to arrival at the hospital ED were not available in the NSHRI dataset. There have been some estimations for the time of self-harm versus the self-harm presentation, such as an estimation of four hours between an overdose and the time at which the patient arrives at an ED [41]. In addition, there was no data on the circumstances of the self-harm presentations. The time of a self-harm presentation to an ED only gives an approximation of the time when the self-harm act occurred, and can be influenced by many factors.

### Further research

Given that much of the research from this study justifies the recommendations made in the NCPSH [9], as previously discussed, it would be imperative for future research to examine to what extent the recommendations have been implemented, and explore the barriers to implementation.

Studies examining the time of the self-harm act itself and the circumstances surrounding the act across the different age-groups could further inform the research from this study. Feasibility of collecting such data and research into the time of self-harm acts compared to the time of self-harm presentations at EDs should be considered in the future. Such data would be useful for both the design of clinical response protocols and the design of public health interventions.

Future studies could examine if these analyses could inform public health messaging around restricting access to means – encouraging parents to keep medications in locked cabinets, for example.

Self-harm presentations involving alcohol are more likely out-of-hours [27] and the proportions of young adults and adults not receiving a mental health assessment following their self-harm presentation were also higher out-of-hours, albeit not substantially. Therefore, we conjectured that alcohol involvement may be an important factor contributing to whether a mental health assessment is carried out or not. Further studies to examine reasons why some self-harm patients do not receive mental health assessments at EDs, using qualitative explorations with patients and clinicians or otherwise, could help inform service delivery for self-harm patients.

Self-harm presentations during the COVID-19 pandemic (i.e. 2020 NSHRI data) were excluded for this study since it this could have skewed the data – it being a period that was not reflective of the typical trends at EDs. While examining this data was not the focus of this study, further research could compare the same trends found in this study to those during and post the COVID-19 period to understand the effect of the pandemic.

## Conclusion

All patients presenting to EDs with a self-harm, regardless of the time, should be given a mental health assessment and referred for appropriate care in order to avoid a repeat self-harm presentation or, indeed, a later death by suicide. Hospitals should ensure that adequate resources are available for individuals presenting with self-harm, especially in the case of overcrowded EDs, and protocols need to be designed for those presenting with self-harm due to intoxication. In line with national policy, pathways for self-harm patients should be designed that can incorporate services from allied health multidisciplinary teams, social work, addiction services and counselling organisations. Future research should examine to what extent such pathways been implemented, and explore the barriers to implementation. Future research should properly investigate other factors, besides alcohol, that lead to some self-harm presenting patients leaving ED without a mental health assessment, and what procedures could be put in place for such patients. Given the lower rates of self-harm during school holidays for children and adolescents, the school environment must be considered in the context of mental health and self-harm public health prevention interventions.

### Electronic supplementary material

Below is the link to the electronic supplementary material.

Supplementary Material 1

## Data Availability

The dataset analysed during the current study are not publicly available due to General Data Protection Regulation (GDPR) but are available from the second author on reasonable request.

## Abbreviations

CAMHS: Child and adolescent mental health services
ED: Emergency Department
NCPSH: National Clinical Programme for Self-Harm and Suicide-related Ideation
NSRF: National Suicide Research Foundation
NSHRI: National Self-Harm Registry Ireland

## Declarations

### Patient and public involvement

No patient and public involvement.
