# Supplementary material for: Comparing times of self-harm presentations to hospital emergency departments in children, adolescents, young adults and adults: a national registry study 2007–2019
Source: BMC Psychiatry. 2024 Jun 27;24:474. doi: 10.1186/s12888-024-05921-x (PMC11210019; doi:10.1186/s12888-024-05921-x)
Supplement: Supplementary file 1 — Supplementary Material 1 [file 12888_2024_5921_MOESM1_ESM.docx]

| **Supplementary Table S1: Sex stratification for the number and percentage of individuals across four age groups by patient sex, time of day, out-of-hours timeframe , weekday and month of self-harm presentation, type of self-harm presentation (method and whether the presentation was a repeat presentation within the time frame)** | | | | | | | | | | |
| --- | --- | --- | --- | --- | --- | --- | --- | --- | --- | --- |
|  | **Males** | | | | | **Females** | | | | |
|  | **Children**  (age 8-12) | **Adolescents**  (age 13-17) | **Young Adults**  (age 18-25) | **Adults**  (age >25) | **Total**  (all ages) | **Children**  (age 8-12) | **Adolescents**  (age 13-17) | **Young Adults**  (age 18-25) | **Adults**  (age >25) | **Total**  (all ages) |
|  |  |  |  |  |  |  |  |  |  |  |
|  | **n (%)** | **n (%)** | **n (%)** | **n (%)** | **n (%)** | **n (%)** | **n (%)** | **n (%)** | **n (%)** | **n (%)** |
|  |  |  |  |  |  |  |  |  |  |  |
| **Repeat self-harm presentations** |  |  |  |  |  |  |  |  |  |  |
| Yes | 12 (4.3) | 1,010 (21.0) | 7,012 (37.1) | 19,698 (43.2) | 27,732 (39.9) | 28 (7.4) | 3,110 (26.4) | 7,914 (39.4) | 23,358 (46.1) | 34,410 (41.5) |
| No | 265 (95.7) | 3,810 (79.0) | 11,888 (62.9) | 25,872 (56.8) | 41,835 (60.1) | 351 (92.6) | 8,657 (73.6) | 12,175 (60.6) | 27,314 (53.9) | 48,497 (58.5) |
|  |  |  |  |  |  |  |  |  |  |  |
| **Time of Presentation** |  |  |  |  |  |  |  |  |  |  |
| 00:00 – 03:59 | 20 (7.2) | 1,049 (21.8) | 4,592 (24.3) | 9,465 (20.8) | 15,126 (21.7) | 47 (12.4) | 2,211 (18.8) | 4,327 (21.5) | 10,184 (20.1) | 16,769 (20.2) |
| 04:00 – 07:59 | <5 (<2.0) | 317 (6.6) | 2,675 (14.2) | 4,321 (9.5) | 7,316 (10.5) | 7 (1.8) | 536 (4.6) | 2,473 (12.3) | 4,579 (9.0) | 7,595 (9.2) |
| 08:00 – 11:59 | 38 (13.7) | 414 (8.6) | 1,679 (8.9) | 4,177 (9.2) | 6,307 (9.1) | 39 (10.3) | 1,044 (8.9) | 1,590 (7.9) | 4,669 (9.2) | 7,342 (8.9) |
| 12:00 – 15:59 | 68 (24.5) | 771 (16.0) | 2,598 (13.7) | 7,635 (16.8) | 11,072 (15.9) | 91 (24.0) | 1,950 (16.6) | 3,208 (16.0) | 8,951 (17.7) | 14,200 (17.1) |
| 16:00 – 19:59 | 76 (27.4) | 1,006 (20.9) | 3,292 (17.4) | 9,284 (20.4) | 13,658 (19.6) | 96 (25.3) | 2,655 (22.6) | 4,029 (20.1) | 10,781 (21.3) | 17,561 (21.2) |
| 20:00 – 23:59 | 72 (26.0) | 1,263 (26.2) | 4,064 (21.5) | 10,688 (23.5) | 16,087 (23.1) | 99 (26.1) | 3,371 (28.6) | 4,462 (22.2) | 11,508 (22.7) | 19,440 (23.4) |
|  |  |  |  |  |  |  |  |  |  |  |
| **In-hours versus out-of-hours** |  |  |  |  |  |  |  |  |  |  |
| In-hours (09:00-17:00, Monday to Friday) | 106 (38.3) | 1,088 (22.6) | 3,357 (17.8) | 9,781 (21.5) | 14,332 (20.6) | 122 (32.2) | 2,711 (23.0) | 4,017 (20.0) | 11,480 (22.7) | 18,330 (22.1) |
| Out-of-hours | 171 (61.7) | 3,732 (77.4) | 15,543 (82.2) | 35,789 (78.5) | 55,235 (79.4) | 257 (67.8) | 9,056 (77.0) | 1,6072 (80.0) | 39,192 (77.3) | 64,577 (77.9) |
|  |  |  |  |  |  |  |  |  |  |  |
| **Weekday of Presentation** |  |  |  |  |  |  |  |  |  |  |
| Monday | 44 (15.9) | 806 (16.7) | 2,734 (14.5) | 6,876 (15.1) | 10,460 (15.0) | 60 (15.8) | 2,121 (18.0) | 3,196 (15.9) | 7,830 (15.5) | 13,207 (15.9) |
| Tuesday | 45 (16.2) | 727 (15.1) | 2,631 (13.9) | 6,410 (14.1) | 9,813 (14.1) | 58 (15.3) | 1,829 (15.5) | 2,855 (14.2) | 6,907 (13.6) | 11,649 (14.1) |
| Wednesday | 47 (17.0) | 691 (14.3) | 2,390 (12.6) | 6,195 (13.6) | 9,323 (13.4) | 66 (17.4) | 1,692 (14.4) | 2,593 (12.9) | 6,820 (13.5) | 11,171 (13.5) |
| Thursday | 46 (16.6) | 700 (14.5) | 2,509 (13.3) | 6,534 (14.3) | 9,789 (14.1) | 62 (16.4) | 1,636 (13.9) | 2,614 (13.0) | 6,838 (13.5) | 11,150 (13.4) |
| Friday | 45 (16.2) | 612 (12.7) | 2,600 (13.8) | 6,492 (14.2) | 9,749 (14.0) | 53 (14.0) | 1,436 (12.2) | 2,564 (12.8) | 6,991 (13.8) | 11,044 (13.3) |
| Saturday | 21 (7.6) | 597 (12.4) | 2,805 (14.8) | 6,435 (14.1) | 9,858 (14.2) | 38 (10.0) | 1,348 (11.5) | 2,806 (14.0) | 7,241 (14.3) | 11,433 (13.8) |
| Sunday | 29 (10.5) | 687 (14.3) | 3,231 (17.1) | 6,628 (14.5) | 10,575 (15.2) | 42 (11.1) | 1,705 (14.5) | 3,461 (17.2) | 8,045 (15.9) | 13,253 (16.0) |
|  |  |  |  |  |  |  |  |  |  |  |
| **Month of Presentation** |  |  |  |  |  |  |  |  |  |  |
| January | 24 (8.7) | 422 (8.8) | 1,557 (8.2) | 3,770 (8.3) | 5,773 (8.3) | 36 (9.5) | 1,162 (9.9) | 1,751 (8.7) | 4,170 (8.2) | 7,119 (8.6) |
| February | 19 (6.9) | 403 (8.4) | 1,521 (8.0) | 3,430 (7.5) | 5,373 (7.7) | 24 (6.3) | 1,013 (8.6) | 1,616 (8.0) | 3,909 (7.7) | 6,562 (7.9) |
| March | 29 (10.5) | 470 (9.8) | 1,652 (8.7) | 3,845 (8.4) | 5,996 (8.6) | 63 (16.6) | 1,096 (9.3) | 1,819 (9.1) | 4,179 (8.2) | 7,157 (8.6) |
| April | 16 (5.8) | 471 (9.8) | 1,470 (7.8) | 3,651 (8.0) | 5,608 (8.1) | 33 (8.7) | 1,060 (9.0) | 1,657 (8.2) | 4,192 (8.3) | 6,942 (8.4) |
| May | 28 (10.1) | 444 (9.2) | 1,657 (8.8) | 4,029 (8.8) | 6,158 (8.9) | 35 (9.2) | 1,106 (9.4) | 1,790 (8.9) | 4,524 (8.9) | 7,455 (9.0) |
| June | 29 (10.5) | 328 (6.8) | 1,618 (8.6) | 3,798 (8.3) | 5,773 (8.3) | 25 (6.6) | 788 (6.7) | 1,701 (8.5) | 4,374 (8.6) | 6,888 (8.3) |
| July | 19 (6.39) | 352 (7.3) | 1,684 (8.9) | 4,251 (9.3) | 6,306 (9.1) | 18 (4.7) | 771 (6.6) | 1,688 (8.4) | 4,563 (9.0) | 7,040 (8.5) |
| August | 19 (6.9) | 352 (7.3) | 1,605 (8.5) | 3,994 (8.8) | 5,970 (8.6) | 19 (5.0) | 797 (6.8) | 1,690 (8.4) | 4,657 (9.2) | 7,163 (8.6) |
| September | 21 (7.6) | 398 (8.3) | 1,501 (7.9) | 3,822 (8.4) | 5,742 (8.3) | 27 (7.1) | 992 (8.4) | 1,500 (7.5) | 4,226 (8.3) | 6,745 (8.1) |
| October | 29 (10.5) | 401 (8.3) | 1,606 (8.5) | 4,012 (8.8) | 6,048 (8.7) | 39 (10.3) | 1,035 (8.8) | 1,677 (8.3) | 4,307 (8.5) | 7,058 (8.5) |
| November | 25 (9.0) | 417 (8.7) | 1,520 (8.0) | 3,450 (7.6) | 5,412 (7.8) | 34 (9.0) | 1,093 (9.3) | 1,655 (8.2) | 3,870 (7.6) | 6,652 (8.0) |
| December | 19 (6.9) | 362 (7.5) | 1,509 (8.0) | 3,518 (7.7) | 5,408 (7.8) | 26 (6.9) | 854 (7.3) | 1,545 (7.7) | 3,701 (7.3) | 6,126 (7.4) |
|  |  |  |  |  |  |  |  |  |  |  |
| **Method of self-harm** |  |  |  |  |  |  |  |  |  |  |
| Drug overdose only | 52 (18.8) | 2,211 (45.9) | 8,892 (47.0) | 25,204 (55.3) | 36,358 (52.3) | 132 (34.8) | 7027 (59.7) | 12,089 (60.2) | 35,955 (71.0) | 55,203 (66.6) |
| Self-cutting only | 73 (26.4) | 1,227 (25.5) | 4,538 (24.0) | 8,607 (18.9) | 14,445 (20.8) | 152 (40.1) | 2624 (22.3) | 4,239 (21.1) | 7,061 (13.9) | 14,076 (17.0) |
| Overdose & self-cutting | <5 (<10.0) | 264 (5.5) | 1,206 (6.4) | 1,853 (4.1) | 3,323 (4.8) | 13 (3.4) | 767 (6.5) | 1,272 (6.3) | 1,867 (3.7) | 3,919 (4.7) |
| Attempted hanging only | 85 (30.7) | 431 (8.9) | 1,343 (7.1) | 2,972 (6.5) | 4,831 (6.9) | 24 (6.3) | 301 (2.6) | 548 (2.7) | 1,199 (2.4) | 2,072 (2.5) |
| Attempted drowning only | 5 (10.0) | 52 (1.1) | 467 (2.5) | 1,436 (3.2) | 1,960 (2.8) | 0 (0.0) | 80 (0.7) | 355 (1.8) | 932 (1.8) | 1,367 (1.6) |
| Other | 60 (21.7) | 635 (13.2) | 2,454 (13.0) | 5,498 (12.1) | 8,647 (12.4) | 58 (15.3) | 968 (8.2) | 1,586 (7.9) | 3,658 (7.2) | 6,270 (7.6) |
|  |  |  |  |  |  |  |  |  |  |  |
| **Mental health assessment conducted (n=81,481)*** |  |  |  |  |  |  |  |  |  |  |
| Yes | 125 (62.8) | 1,822 (65.0) | 6,226 (63.3) | 15,146 (63.5) | 23,319 (63.5) | 210 (77.2) | 5,210 (70.5) | 7,496 (69.2) | 17,804 (67.7) | 30,720 (68.6) |
| No | 41 (20.6) | 688 (24.5) | 2,457 (25.0) | 6,173 (25.9) | 9,359 (25.5) | 39 (14.3) | 1,647 (22.3) | 2,280 (21.0) | 6,051 (23.0) | 10,017 (22.4) |
| Refused | <5 (<10.0) | 41 (1.5) | 349 (3.5) | 861 (3.6) | 1,253 (3.4) | 0 (0.0) | 85 (1.2) | 380 (3.5) | 945 (3.6) | 1,410 (3.1) |
| Unknown | 31 (15.6) | 252 (9.0) | 805 (8.2) | 1,679 (8.2) | 2,767 (7.5) | 23 (8.5) | 444 (6.0) | 677 (6.2) | 1,492 (5.7) | 2,636 (5.9) |
|  |  |  |  |  |  |  |  |  |  |  |
| **Admission details** |  |  |  |  |  |  |  |  |  |  |
| Admitted to a ward | 110 (39.7) | 1,414 (29.3) | 3,565 (18.9) | 11,706 (25.7) | 16,795 (24.1) | 202 (53.3) | 4,650 (39.5) | 4,387 (21.8) | 14,713 (29.0) | 23,952 (28.9) |
| Admitted to psychiatry | <5 (<10.0) | 165 (3.4) | 1,631 (8.6) | 4,917 (10.8) | 6,716 (9.7) | <5 (<1.3) | 284 (2.4) | 1,496 (7.4) | 5,022 (9.9) | 6,806 (8.2) |
| Refused admission or left against  medical advice | <5 (<10.0) | 394 (8.2) | 3,097 (16.4) | 7,910 (17.4) | 11,405 (16.4) | 5 (1.3) | 590 (5.0) | 2,660 (13.2) | 6,896 (13.6) | 10,151 (12.2) |
| Not admitted | 160 (57.8) | 2,847 (59.1) | 10,607 (56.1) | 21,037 (46.2) | 34,651 (49.8) | 168 (44.3) | 6,243 (53.1) | 11,546 (57.5) | 24,041 (47.4) | 41,998 (50.7) |
|  |  |  |  |  |  |  |  |  |  |  |

| **Supplementary Table S2:** **Numbers and percentages for each age group comparing data on mental health assessments of self-harm presentations out-of-hours and in-hours (09:00 – 17:00, Monday – Friday) in EDs from 2007-2019** | | | | |
| --- | --- | --- | --- | --- |
|  | **In-hours**  **n (%)** | **Out-of-hours**  **n (%)** | **p-value** | **Effect size**  **Φ** |
| **Mental health assessment conducted** ^ζ^ |  |  |  |  |
| **Children** |  |  |  |  |
| Yes | 133 (73.1) | 202 (69.9) | 0.570^θ^ | 0.03 |
| No | 29 (15.9) | 51 (17.6) |  |  |
| Refused | <5 (<3.0) | <5 (<3.0) |  |  |
| Unknown | 19 (10.4) | 35 (12.1) |  |  |
| **Adolescents** |  |  |  |  |
| Yes | 1,773 (70.5) | 5,259 (68.5) | 0.161^θ^ | 0.02 |
| No | 555 (22.1) | 1,780 (23.2) |  |  |
| Refused | 14 (0.6) | 112 (1.5) |  |  |
| Unknown | 173 (6.9) | 523 (6.8) |  |  |
| **Young adults** |  |  |  |  |
| Yes | 3,036 (71.1) | 10,686 (65.2) | <0.001 ^θ^ | 0.05 |
| No | 848 (19.8) | 3,889 (23.7) |  |  |
| Refused | 11 (2.6) | 618 (3.8) |  |  |
| Unknown | 278 (6.5) | 1,204 (7.3) |  |  |
| **Adults** |  |  |  |  |
| Yes | 8,168 (69.3) | 24,782 (64.6) | <0.001 ^θ^ | 0.04 |
| No | 2,571 (21.8) | 9,653 (25.2) |  |  |
| Refused | 293 (2.5) | 1,513 (3.9) |  |  |
| Unknown | 758 (6.4) | 2,413 (6.3) |  |  |
|  |  |  |  |  |

**Notes:** In-hours are 09:00 – 17:00, Monday – Friday; **^ζ^** Data collected since 2013. ^θ^ chi-square test within the age-group for yes/no only; the effect size here is the Φ coefficient
